# Rapid Long-Range Disynaptic Inhibition Explains the Formation of Cortical Orientation Maps

**DOI:** 10.3389/fncir.2017.00021

**Published:** 2017-03-31

**Authors:** Ján Antolík

**Affiliations:** Unité de Neurosciences, Information et Complexité, Centre National de la Recherche Scientifique UPR 3293Gif-sur-Yvette, France

**Keywords:** primary visual cortex, rate model, cortical functional development, Hebbian learning, cortical horizontal connectivity, orientation map

## Abstract

Competitive interactions are believed to underlie many types of cortical processing, ranging from memory formation, attention and development of cortical functional organization (e.g., development of orientation maps in primary visual cortex). In the latter case, the competitive interactions happen along the cortical surface, with local populations of neurons reinforcing each other, while competing with those displaced more distally. This specific configuration of lateral interactions is however in stark contrast with the known properties of the anatomical substrate, i.e., excitatory connections (mediating reinforcement) having longer reach than inhibitory ones (mediating competition). No satisfactory biologically plausible resolution of this conflict between anatomical measures, and assumed cortical function has been proposed. Recently a specific pattern of delays between different types of neurons in cat cortex has been discovered, where direct mono-synaptic excitation has approximately the same delay, as the combined delays of the disynaptic inhibitory interactions between excitatory neurons (i.e., the sum of delays from excitatory to inhibitory and from inhibitory to excitatory neurons). Here we show that this specific pattern of delays represents a biologically plausible explanation for how short-range inhibition can support competitive interactions that underlie the development of orientation maps in primary visual cortex. We demonstrate this statement analytically under simplifying conditions, and subsequently show using network simulations that development of orientation maps is preserved when long-range excitation, direct inhibitory to inhibitory interactions, and moderate inequality in the delays between excitatory and inhibitory pathways is added.

## 1. Introduction

**Figure 1:**
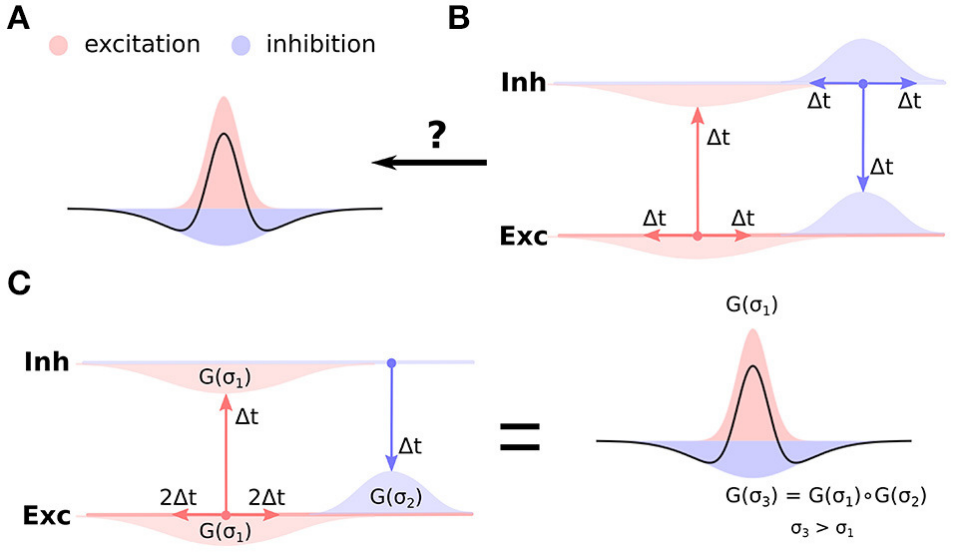
**Cortical anatomy and effective lateral interactions. (A)** Short-range excitation (red) and long-range inhibition (blue) leads to effective Mexican-hat lateral interactions (black curve). **(B)** Anatomical evidence indicates the opposite organization of lateral connectivity in cortex, whereby excitatory neurons send long-range connections to other neurons, while inhibitory neurons only local ones. Furthermore, uniform delays across the intra-cortical projections is typically assumed. It is not clear how such anatomical configuration can support the Mexican-hat effective interaction across cortical surface. **(C)** Under the assumption of slow excitatory-to-excitatory and fast excitatory-to-inhibitory and inhibitory-to-excitatory pathway (and disregarding the inhibitory-to-inhibitory interactions), the effective lateral inhibitory interactions correspond to the convolution of the excitatory-to-inhibitory and inhibitory-to-excitatory connection kernels. Under the assumption that these kernels are Gaussian (G(σ) in the figure), the effective inhibitory interactions will be Gaussian with variance (space-constant) greater then the excitatory-to-excitatory one, thus forming Mexican-hat lateral interaction profile.

Competition between populations of neurons has been proposed as one of the canonical computations of cortical networks, and has been hypothesized to underly a range of brain functions including working memory (Amit and Brunel, 1995; Durstewitz et al., 2000), orientation tuning (Ben-Yishai et al., 1995; Somers et al., 1995), and functional map development (von der Malsburg, 1973; Miikkulainen et al., 2005; Antolík and Bednar, 2011). In developmental models of functional cortical organization the competition occurs between populations of neurons spatially offset along the cortical surface, whereby local populations mutually reinforce each other via excitatory connections (short-range excitation), while long-range inhibition facilitates competition between the local populations and stabilizes the activity in the network. Such so called Mexican-hat arrangement of recurrent interactions (Figure 1A), however, is in stark contrast to the know anatomical arrangement of cortical circuitry: the excitatory neurons (especially in superficial layers) tend to form long-range arborizations spanning multiple columns, while the axons of a majority of inhibitory neurons are confined to an area only several hundreds of micrometers in diameter (Budd and Kisvárday, 2001; Buzás et al., 2006) (Figure 1B).

As pointed out by Muir et al. in their recent study (Muir and Cook, 2014), to solve this apparent conflict, previous topologically organized models of cortical competition have either relied on the anatomically unsupported Mexican-hat profile of lateral interactions (von der Malsburg, 1973; Miikkulainen et al., 2005), or relied on other biologically unrealistic properties, such as selective targeting of inhibitory neurons by long range excitatory connections (Law, 2009; Rutishauser et al., 2012) or instantaneous synaptic transmission coupled with omission of recurrent inhibition (Kang et al., 2003; Grabska-Barwinska and von der Malsburg, 2008; Levy and Reyes, 2011).

A common hypothesis about how to reconcile Mexican-hat lateral interactions with anatomical reality is that the range of the effective inhibitory influence of an excitatory neuron onto other excitatory neurons (via the disynaptic pathway from excitatory to inhibitory to excitatory neurons—the disynaptic inhibition) will correspond to the cumulative reach of the direct excitatory and the direct inhibitory connections. The effective inhibitory interactions between excitatory neurons will thus have longer range than the direct excitatory connections, supporting the use of Mexican-hat lateral interactions in population models (i.e., models without explicit separation of excitatory and inhibitory populations). Even though very intuitive, this explanation has never been explicitly demonstrated, and it omits the fact that under the reasonable null-hypothesis of equal transmission delays for all connections, the recurrent disynaptic inhibition will lag the direct recurrent excitation. As it turns out this is crucial, as recent model analysis by Muir et al. (Muir and Cook, 2014) shows that, under the assumption of uniform transmission delays, the presence of competition across the cortical surface is predicted well by the anatomy of direct excitatory and inhibitory coupling and that multi-synaptic network effects are negligible, effectively rejecting the disynaptic explanation behind Maxican-hat interaction. In conclusion, currently no satisfactory explanation of how topological functional organization develops in cortical networks that is consistent with the present anatomical findings exists.

As we will show in this study, the nature of the transmission delays between different neural type (excitatory, inhibitory) combinations holds the key to resolving this long standing open question. A recent study by Ohana et al. (2012) revealed a specific pattern of transmission delays between different neural type combinations. Specifically they found that the excitatory to excitatory connections are slow, while the excitatory to inhibitory connections are fast. In this study we explore the possibility that this specific transmission delay pattern is the missing link that can explain how short range inhibition can lead to effective cortical competition. We employ computational models to show that the fast excitatory-to-inhibitory-to-excitatory pathway allows for the disynaptic inhibition to generate the effective Mexican-hat like lateral interactions (Figure 1C), thus for the first time explaining competition in topologically organized cortical networks with no biologically implausible assumptions.

To demonstrate the proposed implementation of competitive interactions on a specific feature of the V1 processing, in this study we focus on models of development of functional cortical organization. Functional properties of neurons in primary visual cortex, such orientation, color and frequency preference is not randomly distributed along the cortical surface but rather form smooth topological maps (Swindale, 1996; Goodhill, 2007), whereby nearby neurons prefer similar stimulus features. Such smooth topological mapping of function on cortical surface is ubiquitous throughout the cortex (Huth et al., 2016), found in multiple species, and implicated in a number of functional properties of V1, and of cortex in general. Here we restrict our attention to orientation preference, as it is the most well explored example of competition driven functional cortical organization development, but these results generalize to development of other cortical topological properties, as well as potentially other competition based computations.

## 2. Materials and methods

Here we describe the two models and their variants used in this study and finish with a description of a measure for assessing the extent to which model orientation maps resemble their biological counterparts. Since this study heavily leans on methodology developed in our previous studies, we offer here a short description and refer readers to the original articles for the details.

### 2.1. GCAL model

In Section 3.1 through Section 3.3 (model 1, 2 and 3; see Figure 3) we use the GCAL model (Stevens et al., 2013) which is the most advanced variant of the LISSOM (Laterally Interconnected Synergetically Self-Organizing Map) algorithm introduced by Miikkulainen et al. (2005), which itself is based on earlier Self-Organizing Map models (Kohonen, 1982). Several mechanistic explanations of how orientation maps can develop in primary visual cortex have been proposed, but most, including the LISSOM family of models, involve two key ingredients: (1) stimulus driven Hebbian learning on the thalamo-cortical synapses, that ensures the formation of afferent connectivity pattern inducing Gabor like RFs (and consequently orientation, frequency and phase preference) to V1 neural units; and (2) a Mexican-hat-like effective lateral interactions within the cortical population of neural units, that induce co-activation among proximate units while competition between more distal units.

To understand how these two mechanisms lead to development of orientation maps, consider first the initial state of the model with isotropic afferent connectivity. When stimulus is presented to the model for the first time, the activity of the cortical population will after few simulation steps settle into a pattern that can be best described as random placement of “blobs,” whereby nearby neurons tend to be either co-active or silent. At this point, the placement of the local activity centers (the “blobs”) is essentially random, determined by the interplay of the competitive influence of the lateral interactions with whatever source of variability present in the model (e.g., random noise in the initial connections or content of the stimulus). The Hebbian learning will ensure, that all neurons within the active local populations will adjust they afferent weights slightly toward the stimulus pattern appearing within their RFs. They will thus in subsequent iterations be slightly more activated by similar patterns.

This iterative process of stimulus presentation, activity pattern formation and Hebbian adjustment of the thalamo-cortical weights will keep repeating. However note, that in the subsequent iterations, neurons whose afferent weights will be more similar with the currently presented stimulus falling within their RF will tend to be more active then those whose RF at that point differs from the presented stimulus. Importantly even small differences of the initial activations will be magnified by the lateral competitive interactions thus overall driving nearby neurons to over time develop sharp selectivity for similar stimulus features while more distal neurons will be driven to develop selectivity to other stimulus features. Which features will be mapped onto cortical surface will depend on the exact statistics of the stimuli shown during training. It turns out that if the stimuli are natural images, or other artificial stimuli with strong oriented components, smooth representation of orientation preference across the cortical surface (the orientation maps) will develop. In this study we will focus on this specific feature (orientation), as it is the most well studied one, but it is important to emphasize that given the right stimulation conditions, multiplexed representation of other features, such as ocular dominance, disparity, spatial frequency and others can develop (Miikkulainen et al., 2005).

**Figure 2:**
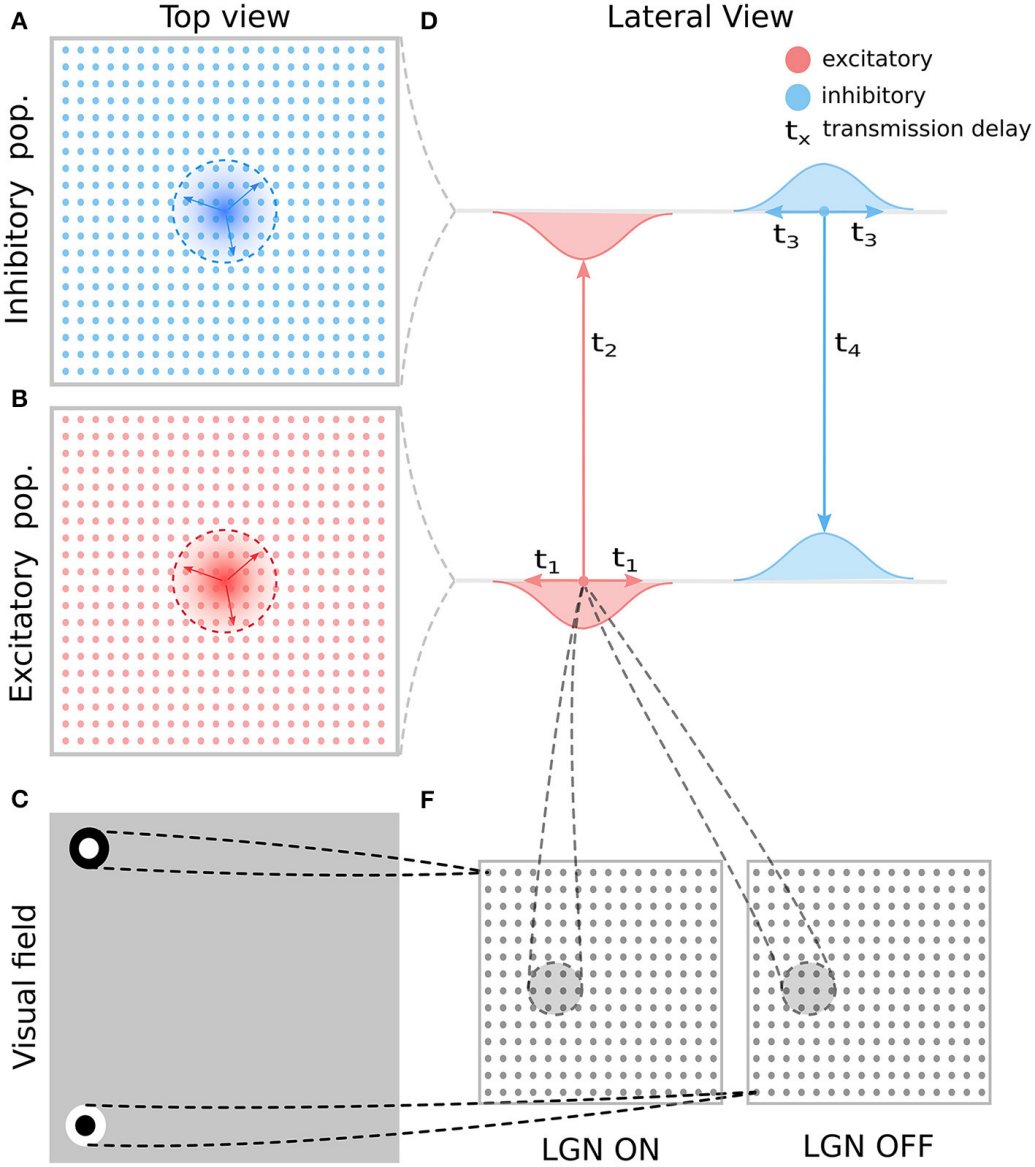
**General model architecture. (A)** The inhibitory cortical population corresponds to a regular lattice of units in cortical space. **(B)** As in A but for the excitatory population. **(C)** Thalamic neurons are modeled as simple difference-of-Gaussian filters followed by a threshold-linear transfer function. **(D)** The intra-cortical connectivity between the excitatory and inhibitory populations and their transmission delays. The intra-cortical connectivity is the only aspect of the general architecture presented here that changes between the different model variants explored in this study and each variant is detailed in Figure 3. **(E)** The RF centers of the LGN neurons form a regular latice across the visual space covered by the model. This retinotopic mapping of connection fields between layers of the model is also maintained in the thalamo-cortical projection (lines between **D,E**). These thalamo-cortical projections are the only connections in the model that undergo Hebbian adaptation during simulated visually driven development.

**Figure 3:**
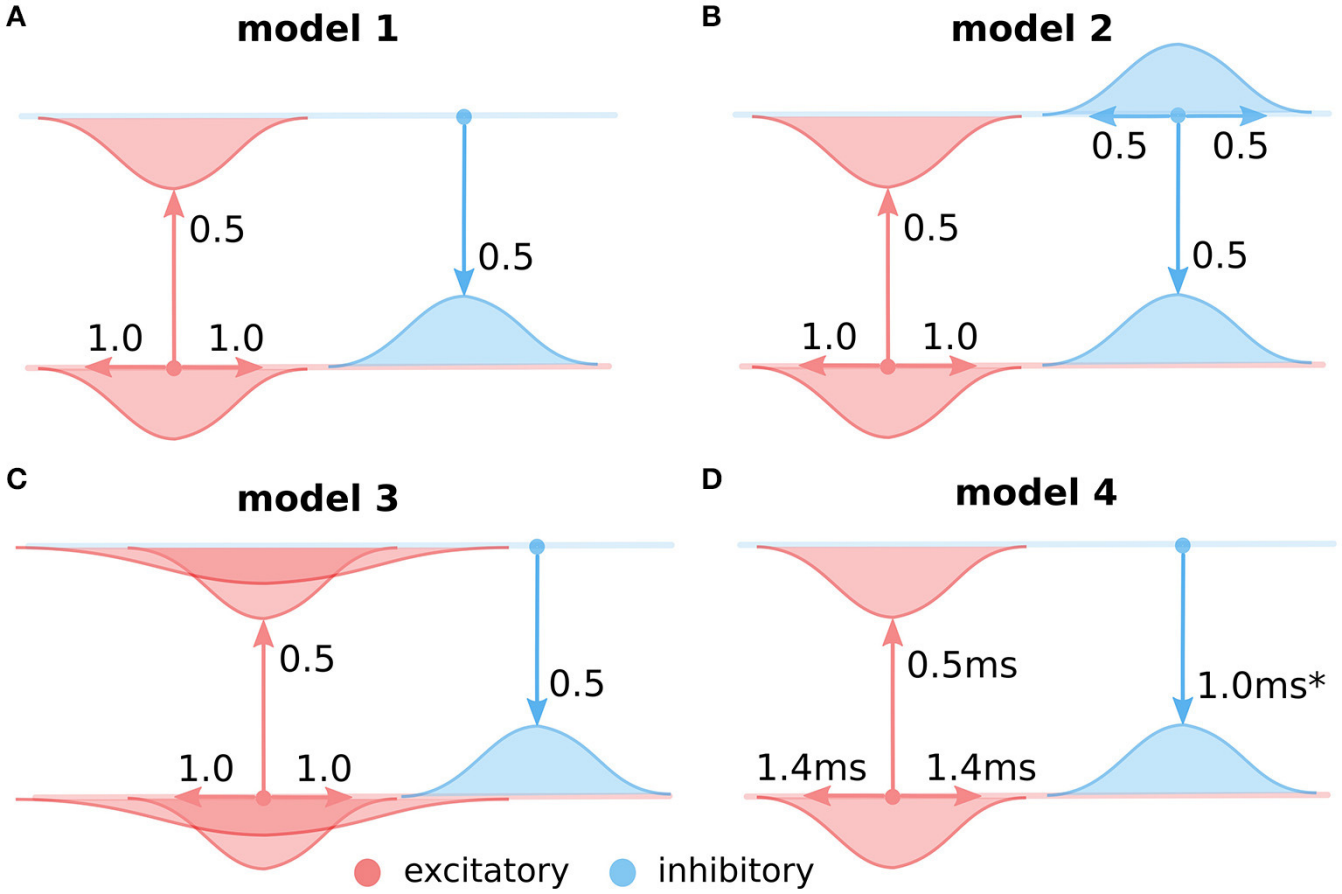
**The four considered intra-cortical connectivity variants**. As in Figure 2, in all panels, the top line represents the excitatory and the bottom line the inhibitory population of neurons. Models **(A–C)** are using firing rate models of neural units with instantaneous translation of inputs into membrane-potential (see Section 2.1). **(A)** Variant 1, assuming only local equally wide excitatory and inhibitory connectivity, and ignoring direct inhibitory to inhibitory interactions. **(B)** Variant 2, as in **(A)** but with added direct inhibitory to inhibitory connections of equal extent as the other excitatory and inhibitory projections. **(C)** Variant 3, as in **(A)** but with added long-range excitatory connections represented based on Buzás et al. (2006) as a second wider Gaussian. **(D)** The same as **(A)** but modeled with neural units that take into account membrane time-constant (see Section 2.2). The transmission delay of the inhibitory to excitatory projections in model 4 was varied in the experiments presented in Section 3.4 (Figure 7).

In the reminder of this section we will describe one specific implementation of model with overall dynamics following the broad description outlined in the previous paragraphs. For clarity and consistency the following model description closely follows the methodology sections in our previous work (Antolík and Bednar, 2011; Stevens et al., 2013). The architecture of the 3 GCAL variants presented in this paper is depicted in Figures 2, 3A–C. The models are implemented in the Topographica simulator, freely available at www.topographica.org. Each of the GCAL models consists of a set of sheets of model neural units, corresponding to: (1) the photo-receptors, (2) the combined effects of the retinal ganglion cells (RGC) and ON and OFF LGN cells. Furthermore each model has one V1 sheet of units with direct excitatory and inhibitory interactions (model 1; see Figure 3), or two V1 sheets, one corresponding to excitatory and one to inhibitory neurons (model 2 and 3; see Figure 3). The model sheet is a 2D array of computational elements (called units or, loosely, neurons), with activation and plasticity equations as described below and referenced by a coordinate system we will refer to as sheet coordinates, where the center of the sheet is designated (0.0,0.0). The number of units simulated in each sheet is determined by the density of units per unit length in both sheet dimensions. All cortical sheets have nominal dimensions 1.0 × 1.0 in sheet coordinates. The sizes of the RGC/LGN (1.5 × 1.5) and photo-receptor (3.5 × 3.5) sheets were chosen to ensure that each unit in the receiving sheet has a complete set of connections, thus minimizing edge effects in the RGC/LGN and V1 sheets. The density of units per 1.0 × 1.0 area is 96 × 96 for the photo-receptor and RGC/LGN ON and OFF sheets, and 96 × 96 for both cortical sheets.

#### 2.1.1. Simulation run-time and stimuli

As a simplification, GCAL ignores the detailed temporal properties of the sub-cortical neural responses and of signal propagation along the various types of connections. Instead, the model ON/OFF units have a constant, sustained output, and all connections have a constant delay, independent of the physical length of that connection. The simulator operates in discrete time steps. Retinal input changes every 16 time steps (and during this period is kept constant), and therefore afferent inputs to the V1 sheet(s) are effectively updated every 16 steps. This process is a discrete simulation of an otherwise continuous series of changes in membrane potential due to incoming spikes and consequent generation of spikes. One such training iteration (16 steps) in the model represents one visual fixation i.e., an iteration consists of a constant retinal activation, followed by processing at the ON/OFF and cortical levels.

The activation value ψ_*i*_ of unit i in the photo-receptor sheet (P) is given by the gray-scale value in the chosen image at that point. In this study we use two-dimensional elongated Gaussian patterns, whose center coordinates and orientation are sampled from a uniform distribution that covers the area of the photo-receptor sheet and the full range of orientations. Every 16 time steps 2 Gaussian patterns are superimposed to form the visual input.

#### 2.1.2. The LGN/RGC ON and OFF sheets

The ON/OFF units are called RGC/LGN units because they represent the entire pathway between the retinal photo-receptors and V1, including the retinal ganglion cells, LGN cells, and the connection pathways. The activation level for a unit at position *j* in an RGC/LGN sheet at time *t* is defined as:

(1) $$\eta_{j}(t)=f\left(\frac{A_{j}(t)}{c+\gamma_{L}\sum_{k}A_{k}(t)l_{kj}}\right)$$

where

(2) $$A_{j}(t)=\gamma_{F}\sum_{i\in F_{j}}\Psi_{i}(t)\omega_{ij}$$

The activation function *f* is a half-wave rectifying function that ensures positive activation values, constant γ_*F*_ = 1.5 defines the overall strength of the afferent connections from the retina, constant γ_*L*_ = 0.6 defines the strength of the lateral connections within the RGC/LGN sheet, constant *c* = 0.11 defines the slope of the gain, Ψ_*i*_ is the activation of unit *i* taken from the set of photo-receptors from which RGC/LGN unit *j* receives input (its connection field *F*_*j*_), ω_*ij*_ is the connection weight from unit *i* in the retina to unit *j* in the RGC/LGN, and *l*_*kj*_ is the lateral connection weight from RGC/LGN unit *k* to RGC/LGN unit *j*. Weights from the photo-receptors to units in the ON and OFF channels are set to fixed strengths with a difference-of-Gaussians kernel (σ_*center*_ = 0.07385, σ_*surround*_ = 0.2954, in sheet dimensions), with ON connection fields having a positive center and a negative surround and vice versa for OFF. The lateral RGC/LGN weights are 2D Gaussians with kernel size σ = 0.25. The center of the afferent and lateral connection field of each ON/OFF unit is mapped to the location in the photo-receptor and LGN sheet corresponding to the location of that unit in sheet coordinates, making all these projections retinotopic.

#### 2.1.3. Cortical model

**Figure 4:**
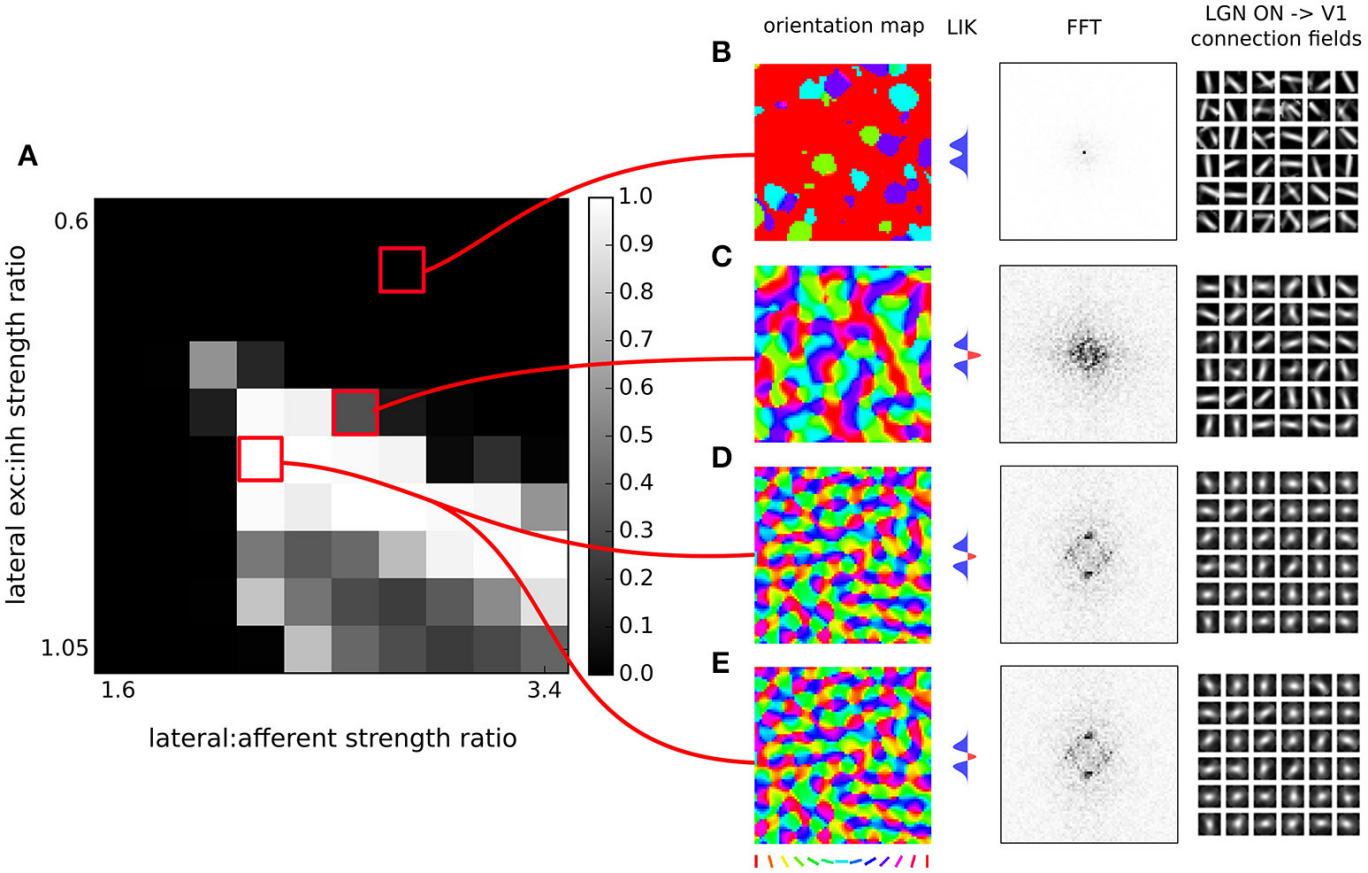
**Orientation map development with short range inhibition and a fast excitatory-to-inhibitory-to-excitatory loop. (A)** Map quality (see Section 2) at a range of lateral excitatory vs. inhibitory projection strength ratios and afferent vs. lateral projection strength ratios. **(B–D)** Functional organization in 3 example parameter configurations indicated by the red marks. From left to right, the orientation map, the lateral interaction kernel (LIK; this is calculated based on Equation 14), fast-Fourier transform of the orientation map and afferent connection fields from the ON LGN model sheet for 25 example model V1 neurons. **(B,C)** Two examples of sub-optimal orientation maps. **(D)** Model configuration with the highest quality map found in this parameter search. **(E)** The same model configuration but in this case it was run with explicit simulation of inhibitory neurons and corresponding connections. **(D,E)** are nearly identical confirming the correctness of our analysis.

Units in the cortical sheets each receive three types of projections represented as matrices of weights: afferent excitatory (*p* = *A*), lateral excitatory (*p* = *E*) and lateral inhibitory (*p* = *I*). The contribution *C*_*jp*_ to the activation of unit *j* in a cortical sheet from each projection *p* at time *t* is given by:

(3) $$C_{jp}(t+\delta t)=\sum_{i\in F_{jp}}\Psi_{i}(t)\omega_{pij}$$

where Ψ_*i*_(*t*) is the activation of unit *i* taken from the set of units in the input sheet of projection *p* from which unit *j* receives input (its connection field *F*_*jp*_), and ω_*pij*_ is the connection weight from unit *i* in the input sheet of projection *p* to unit *j* in the output sheet of projection *p*. All connection field weights are initialized with uniform random noise multiplied by a 2D Gaussian profile, cut off at the distance specified below. Contributions from each projection are weighted and summed and passed via a non-linearity *f* to calculate the activation of a cortical neuron i at time *t*:

(4) $$\Psi_{i}(t)=f(\sum_{p}\gamma_{p}C_{ip}(t))$$

where γ_*p*_ is a constant determining the sign (negative for inhibitory) and strength of projection *p*. The transfer function *f* is a half-wave rectifying function that ensures positive activation values. It has a variable threshold point (θ) dependent on the average activity of the unit as described in the next subsection, but in all cases the gain is fixed at unity. The projection strength scaling factor of the afferent projection γ_*A*_ was set to 1.5 based on Stevens et al. (2013) while the values of the lateral excitatory and inhibitory scaling factors, γ_*E*_ and γ_*I*_ respectively, were varied (see Figure 4) to find a balance between excitation and inhibition, and between afferent and lateral influences, to provide robust formation of activity bubbles that facilitates the formation of smooth maps.

Once all 16 settling steps are complete, the settled cortical activation pattern is deemed to be the response of cortical sheets to the presented pattern. At this point we use the response of cortical neurons to update their threshold point (θ) (using the adaptation process described below) and to update the afferent weights via Hebbian learning. Cortical activity is then reset to zero, and a new pattern is presented. Note that both adaptation and learning could instead be performed at every settling step, but this would greatly decrease computational efficiency.

#### 2.1.4. Homeostatic adaptation

The threshold θ of all cortical excitatory units is updated at the end of each settling phase based on the following equations:

(5) $$\theta_{t\;+\;1}=\theta_{t}+\xi (\tilde{\Psi }(t)-\mu )$$

where ξ = 0.01 is the time constant of the threshold adaptation, μ = 0.24 is a constant defining the target average activity, and $\tilde{\text{Ψ}}$ is the recent average activity of the unit:

(6) $$\tilde{\Psi }(t)=(1-\chi )\Psi (t)+\chi \tilde{\Psi }(t-1)$$

where Ψ(*t*) is the output of the unit at time t and χ = 0.991 is a time constant controlling the decay of the influence of the past activities. The effect of this scaling mechanism is to bring the average activity of each cortical unit closer to the specified target. If the activity in a V1 unit moves away from the target during training, the threshold for activation is thus automatically raised or lowered to bring it closer to the target. Note that an alternative rule with only a single smoothing parameter (rather than ξ and χ) could be formulated, but the rule as presented here makes it simple for the modeler to set a desired target activity.

#### 2.1.5. Hebbian adaptation

The initial connection field weights are isotropic 2D Gaussians for the lateral excitatory projection and uniformly random within a Gaussian envelope for afferent and lateral inhibitory projections. Specifically, a neuron located at (i, j) will have the following weights in projection *p*:

(7) $$\omega_{ijp}=\frac{1}{Z_{p}}uexp(-\frac{x^{2}+y^{2}}{2\sigma_{p}^{2}})$$

where (x, y) is the sheet-coordinate location of the presynaptic neuron, *u* = 1 for the lateral excitatory projection (*p* = E), and u is a scalar value drawn from a uniform random distribution for the afferent and lateral inhibitory projections (*p* = A, I), σ_*p*_ determines the width of the Gaussian in sheet coordinates (σ_*A*_ = 0.27, σ_*E*_ = 0.035, $\sigma_{I}=0.035\sqrt{2}$), and *Z*_*p*_ is a constant normalizing term that ensures that the total of all weights ω_*ijp*_ to neuron *j* in projection *p* is 1.0. Weights for each projection are only defined within a specific maximum circular radius *r*_*p*_ (*r*_*A*_ = 0.27, *r*_*E*_ = 0.15, $r_{I}=0.15\sqrt{2}$).

**Figure 5:**
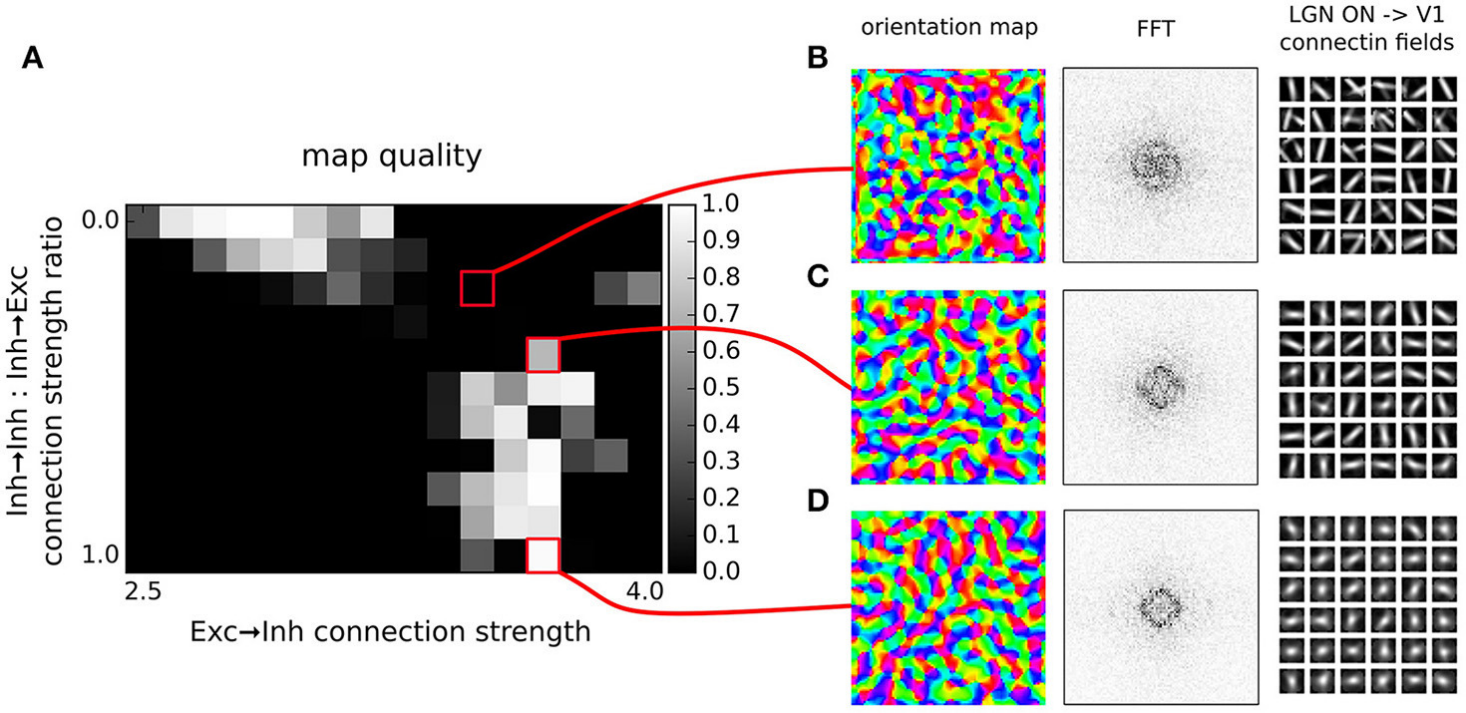
**Orientation map development with short range inhibition, a fast excitatory-to-inhibitory-to-excitatory loop and inhibitory to inhibitory connections**. **(A)** Map quality (see Section 2) at a range of inhibitory to inhibitory vs. inhibitory to excitatory projection strength ratios and excitatory to inhibitory connection strengths. **(B–D)** Functional organization in 3 example parameter configurations indicated by the red marks. From left to right, the orientation map, the lateral interaction kernel (LIK; this is calculated based on Equation 14), fast-Fourier transform of the orientation map and afferent connection fields from the ON LGN model sheet for 25 example model V1 neurons.

**Figure 6:**
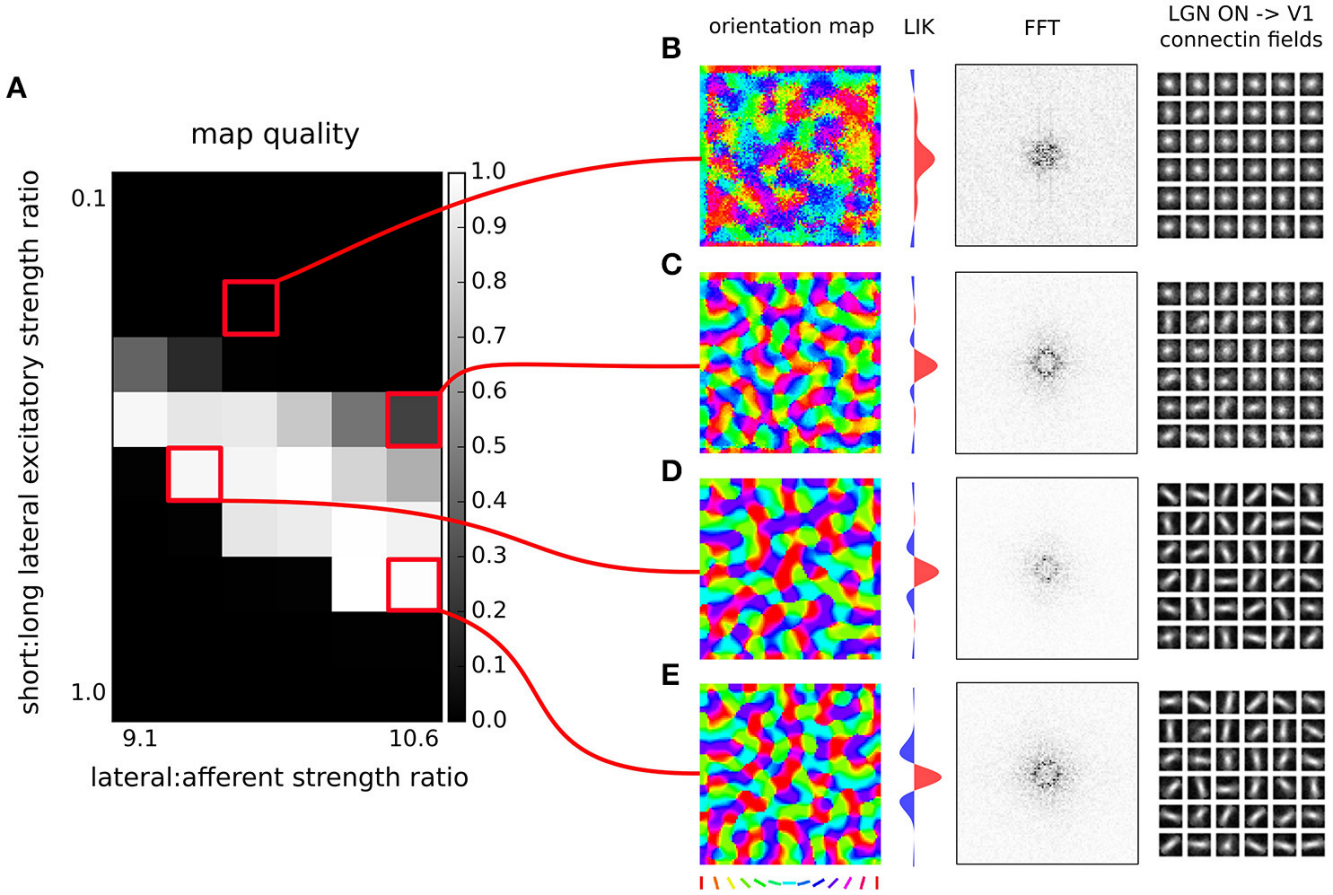
**Orientation map development with short range inhibition, a fast excitatory-to-inhibitory-to-excitatory loop and long-range excitation. (A)** Map quality (see Section 2) at a range of short vs. long range excitatory connection strength ratios (y-axis) and a range of afferent vs. lateral connection strength ratios (x-axis). **(B–E)** Functional organization in 4 example parameter configurations indicated by the red marks. From left to right, the orientation map, fast-Fourier transform of the orientation map and afferent connection fields from the ON LGN model sheet for 25 example model V1 neurons.

In the model, as images are presented to the photo-receptors, the cortical afferent connection weights ω_*i,j,A*_ from the ON/OFF sheets are adjusted once per iteration (after cortical settling is completed) using a simple Hebbian learning rule. This rule results in connections that reflect correlations between the presynaptic ON/OFF unit activities and the postsynaptic cortical response. Hebbian connection weight adjustment at each iteration is dependent on the presynaptic activity, the postsynaptic response, and the Hebbian learning rate:

(8) $$\omega_{ijA}(t)=\frac{\omega_{ij}(t-1)+\beta_{p}\Psi_{j}(t)\Psi_{i}(t)}{\sum_{p\in \{ON,OFF\}}\sum_{k}\left(\omega_{kj,p}(t-1)+\beta_{p}\Psi_{j}(t)\Psi_{k}(t)\right)}$$

where β_*p*_ is the Hebbian learning rate for the connection fields in the two afferent projections from RGC/LGN *p* ∈ {ON, OFF}. i.e., the afferent weights from RGC/LGN are normalized jointly. Learning rate parameters are specified as a fixed value ι_*p*_ = 0.2 for each projection, and then the unit-specific values used in the equation above are calculated as $\beta_{p}=\frac{\iota_{p}}{\upsilon_{p}}$, where υ_*p*_ is the number of connections per connection field in projection *p*. The base parameters described here correspond to the first model variant (Figure 4). Any modifications of these base parameters in the other two GCAL model variants 2 and 3 (Figures 5, 6) examined in Sections 3.2 and 3.3 are then reported in the respective sections.

### 2.2. Rate model with membrane time constant

The architecture of this model (model variant 4; Figures 2, 3D) is identical to the GCAL models (model variants 1 through 3; Figures 3A–C) with the exception of the Equations (3) and (4), which were replaced with an equation taking into account the membrane time constant:

(9) $$\tau_{z}\frac{\delta \Psi_{i}}{\delta t}=-\Psi_{i}+\sum_{p}\gamma_{p}\sum_{j\in F_{ip}}f(\Psi_{j}(t-\nu_{p}))\omega_{pji}$$

where τ_*z*_ (*z* ∈ {*E, I*}) is the membrane time constant of excitatory and inhibitory neurons (τ_*E*_ = 2 ms, τ_*I*_ = 0.5 ms), and ν_*p*_ is the transmission delay for projection *p* (ν_*EE*_ = 1.4 ms, ν_*EI*_ = 0.5 ms,ν_*IE*_ was varied, see Section 3.4). As a consequence the evolution of the network dynamics has to be simulated at higher resolution. We chose the update step to be 0.1 ms, and we let the activity in the model settle for 150 ms, thus resulting in 1,500 settling steps as opposed to the 16 of the GCAL model. The sub-cortical parametrization of the model is identical to the model variants 1–3 (Figures 3A–C), but because the addition of membrane time constants substantially changes the cortical dynamics the cortical parameters had to be re-adjusted. Specifically the strength of the thalamo-cortical connections was set to γ_*A*_ = 0.5, the strength of the lateral excitatory-to-excitatory connections was set to γ_*EE*_ = 3.5, the strength of the lateral excitatory-to-inhibitory connections was set to γ_*EI*_ = 1.0, and the strength of the lateral inhibitory-to-excitatory connections was set to γ_*IE*_ = 2.94.

### 2.3. Orientation map analysis

Model orientation maps are calculated based on the vector average method (Miikkulainen et al., 2005). We first determine the preferred frequency of neurons across the map. Due to the simplified stereotypical stimulus used in this study (elongated Gaussian inputs) the spatial frequency preference of all neurons lies in a very narrow band, and we thus use the mean preferred spatial frequency across all cortical neurons as the value for the spatial frequency parameter across all subsequent analysis. Next, sinusoidal grating inputs that cover the full range of remaining parameter values (combinations of all orientations and phases) are presented, and for each orientation, the peak response of the neuron is recorded. The orientation preference is calculated by constructing a vector for each orientation θ (between 0 and 180°), with the peak response as the length and θ as its orientation. These vectors are summed and the preferred orientation is calculated as half of the orientation of the summed vector. The selectivity is given by the magnitude of the summed vector.

### 2.4. Orientation map quality measure

In order to assess whether the proposed model develops orientation maps that match the structure of those found in real animals we need to utilize an automatic metric that tells how close the maps are to animal data. To this end we will utilize a map-quality metric that we have recently developed (Stevens et al., 2013) based on the empirical observation that pinwheel count in biological orientation maps scales linearly with hypercolumn size across many different species (Kaschube et al., 2010). Specifically the pinwheel density per hypercolumn area (Λ^2^) converges to π, when averaged across a sufficiently large cortical surface. For a detailed description of the procedure for calculating this metric we refer the reader to our previous work (Stevens et al., 2013), but briefly, its calculation involves three steps. First, the locations of the pinwheels in the orientation map are determined as the intersections of the zero contours of the real and imaginary components in the polar representation of the maps, thus yielding the total pinwheel count in the map. Second the hypercolumn size is determined as the peak in the isotropic ring-like Fourier transform of the orientation maps. Third, using these two numbers we can derive the pinwheel density, but to transform it to a useful metric between unity (high-quality map) and zero (low quality map) we pass it through a normalized Gamma distribution. We have shown that this metric reliably distinguishes low and high quality maps (Stevens et al., 2013) and is a valid measure for assessing how well the model orientation maps match animal data.

## 3. Results

In this article we will proceed through multiple gradually more complex models of orientation development, addressing several of the major issues with modeling this phenomenon, eventually demonstrating that the experimentally identified fast inhibitory loop is a satisfactory explanation for how short-range inhibition can support the development of cortical functional organization. We will use two different computational abstractions to explore the questions at hand. In the first part of the study we will use a computational model that is derived from the LISSOM family of models (Miikkulainen et al., 2005). This choice has three advantages. It allows for a very straightforward explanation of why a fast inhibitory loop enables short-range inhibition to induce competition. It makes our explanation directly comparable to the extensive set of published LISSOM family models, and thus demonstrates that the solution proposed here generalizes to a range of other functional properties. And finally the LISSOM abstraction enables very fast simulations, thus allowing us to perform a parameter search analysis that would otherwise be computationally prohibitive. However, as we will explain further in Section 3.4, some simplifications made by the LISSOM abstraction, specifically the instantaneous translation of the neuronal input into its activity, will leave certain questions unanswered. These will be addressed in Section 3.4 using more detailed rate model framework.

### 3.1. A fast excitatory to inhibitory to excitatory loop enables competition in networks with short-range inhibitory connections

Ohana et al. (2012) have shown that transmission delays between different types of cortical neurons are not uniform, specifically they found that on average the transmission delays between excitatory neurons are ~1.4 ms, from excitatory to inhibitory neurons are ~0.5 ms, and from inhibitory to excitatory neurons are ~0.98 ms. The sample size for connections between inhibitory cells in the study was not large enough to be quantitatively reliable. The key observation here is that the combined di-synaptic delay from excitatory to inhibitory and inhibitory to excitatory cells is approximately as long as the mono-synaptic delay of the excitatory to excitatory connections. Drawing from this, the core insight of this study is that under such a pattern of delays the effective inhibitory interactions are well approximated by the convolution of the excitatory and inhibitory connection kernels, as we will show below. This gives the effective inhibition longer range, consequently fulfilling the essential requirement for cortical competition to occur. For didactic purposes, and to simplify analytical treatment, let us first explore one specific set of conditions under which the above statement holds exactly, we will explore the situation when these conditions are relaxed in subsequent sections:

1. The sum of the excitatory to inhibitory and inhibitory to excitatory delays is exactly the same as the excitatory to excitatory delay.
2. There is no inhibitory to inhibitory interaction.
3. The synaptic inputs into the excitatory and inhibitory neurons are instantaneously translated into their rate response via a positive rectified transfer function.
4. The connection kernels of all neurons are Gaussian kernels with spatial constant σ_*e*_ for excitatory neurons and σ_*i*_ for inhibitory neurons.
5. We will assume only local connectivity (disregarding long-range excitatory connections), and assume that both excitatory and inhibitory neurons have the same extent, thus σ_*e*_ = σ_*i*_.

These conditions lead to the following set of equations governing the cortico-cortical interaction.

(10) $$\begin{array}{l} R_{e}(x,t)=[\sum_{y}N_{\sigma_{e}}(\|y-x\|)R_{e}(y,t-\theta_{EE})\\ \;{-N_{\sigma_{i}}(\|y-x\|)R_{i}(y,t-\theta_{IE})+I_{aff}(x,t)]}^{+}\\ R_{i}(x,t)={\left[\sum_{y}N_{\sigma_{e}}(\|y-x\|)R_{e}(y,t-\theta_{EI})\right]}^{+} \end{array}$$

where *R*_*p*_(*x, t*) is the response of a neuron of type *p* located at position *x* at time *t*, *I*_*aff*_(*x, t*) is the afferent input to a neuron at position *x* at time *t*, *N*_σ_ is a normal distribution of variance σ, corresponding to the lateral connection kernel, and θ_*ab*_ is a delay on connections from neural type *a* to neural type *b*. When we expand for *R*_*i*_ we obtain:

(11) $$\begin{array}{l} R_{e}(x,t)=[\sum_{y}N_{\sigma_{e}}(\|y-x\|)R_{e}(y,t-\theta_{EE})-\sum_{y}N_{\sigma_{i}}\\ \;{\left(\|y-x\|)[\sum_{z}N_{\sigma_{e}}(\|z-y\|)R_{e}(z,t-\theta_{EI}-\theta_{IE}\right)]}^{+}\\ \;{+\;I_{aff}(x,t)]}^{+} \end{array}$$

Because θ_*EI*_ + θ_*IE*_ = θ_*EE*_ (assumption 1) and because the inhibitory neurons receive only excitatory connections (assumption 2) we can further simplify the above as:

(12) $$\begin{array}{l} R_{e}(x,t)=[\sum_{y}N_{\sigma_{e}}(\|y-x\|)R_{e}(y,t-\theta_{EE})\\ \;-\sum_{z}\sum_{y}N_{\sigma_{i}}(\|y-x\|)N_{\sigma_{e}}(\|z-y\|)R_{e}(z,t-\theta_{EE})\\ \;+\;I_{aff}(x,t){]}^{+} \end{array}$$

and thus due to the symmetry of the normal distribution:

(13) $$\begin{array}{l} R_{e}(x,t)=[\sum_{y}N_{\sigma_{e}}(\|y-x\|)R_{e}(y,t-\theta_{EE})\\ \;-\sum_{z}N_{\sigma_{i}}*N_{\sigma_{e}}(\|z-x\|)R_{e}(z,t-\theta_{EE})\\ \;+\;I_{aff}(x,t){]}^{+} \end{array}$$

Because convolution of two Normal distributions of variance σ_*i*_ and σ_*e*_ is a normal distribution with variance $\sqrt{\sigma_{i}^{2}+\sigma_{e}^{2}}$ and because σ_*i*_ = σ_*e*_ we can simplify to:

(14) $$\begin{array}{l} R_{e}(x,t)=[\sum_{y}N_{\sigma_{e}}(\|y-x\|)R_{e}(y,t-\theta_{EE})\\ \;-\sum_{z}N_{\sqrt{2}\sigma_{e}}(\|z-x\|)R_{e}(z,t-\theta_{EE})\\ \;+\;I_{aff}(x,t){]}^{+} \end{array}$$

This shows that under these specific assumptions the effective inhibitory interactions between excitatory neurons are $\sqrt{2}$ longer than the excitatory ones and thus follow the Mexican-hat like profile required for the lateral cortical competition underlying functional map development. Even though this essential condition of cortical competition is fulfilled in this model configuration, important constraints on the extent of the effective lateral interactions (relative to lateral excitation) remain. It is thus still unclear whether development of high-quality orientation maps as observed experimentally is supported under these conditions. Conveniently, the lateral interaction in the LISSOM family of models is governed by the same equations as 10. In the following we will use the GCAL model (Stevens et al., 2013), the latest and most robust addition to the LISSOM family of models to demonstrate that the above specific configuration of effective lateral excitation and inhibition permits the development of high-quality orientation maps. GCAL is the only model of stimulus dependent functional development which achieves emergence of biologically realistic orientation maps in terms of pinwheel density, a signature that is a useful objective measure of orientation map quality. We will utilize this map quality measure to reliably detect model configurations which permit the successful emergence of orientation maps. To facilitate a comparison we will use exactly the same model configuration as in Stevens et al. (2013) (see Section 2), except for three modifications necessitated by the analysis above (see Figure 3A):

1. We will change the spread of lateral inhibition to be $\sqrt{2}$ longer than lateral excitatory spread, such that the resulting lateral interactions conform with Equation (14).
2. The change in point 1 will result in a change in the balance of overall excitation and inhibition in the model which is critical for successful functional development in the model. We will thus modify the strength of the lateral inhibition to compensate for changes due to modification 1.
3. Analogously to 2, the changes in 1 change the overall balance between feed-forward and lateral contributions to model a cortical neuron's activity, which we will compensate by changing the overall strength of the lateral interactions.

In order to find a working combination of parameters in points 2 and 3, and also to show that the model is robust to a certain level of changes in these two parameters, we have performed a parameter search across these two parameters and evaluated the quality of the orientation map (see Section 2) for each parameter combination. As Figure 4 shows, under a range of values of both parameters the model develops high-quality orientation maps indistinguishable from their experimental counterparts, thus concluding our first step toward showing that a fast excitatory-to-inhibitory-to-excitatory loop can explain how short-range inhibition can induce cortical competition and consequently the development of topological organization of functional properties.

Furthermore, note that the GCAL model used in Figure 4 only explicitly models excitatory neurons and assumes both direct excitatory and inhibitory interactions between them, thus corresponding to Equation (14). Even though above we have shown that Equation (14) is equivalent to Equation (10) to verify the correctness of our analysis we have run a single simulation of the GCAL model corresponding to the parameter combination with the highest map quality found in Figure 4, but with an explicitly simulated inhibitory population (Figure 4E). In this model we thus do not model direct inhibitory interactions between excitatory neurons, but instead add excitatory to inhibitory and inhibitory to excitatory connections of the same extent as those of the excitatory to excitatory pathway (see the assumption #5). As we have shown above this model should be mathematically equivalent to the simulation shown in Figure 4D. Note, however, that the GCAL simulations represent a discrete approximation in both time and space of Equations (10, 14) and we thus expect small numerical discrepancies. Indeed, orientation maps shown in Figure 4E are nearly identical to those in the Figure 4D, with only barely perceptible numerical differences, confirming the validity of our approach.

### 3.2. Inhibitory to inhibitory connections are consistent with development of functional organization

In the previous section we have shown that under a set of specific assumptions that facilitate analytical treatment, short-range inhibition can induce effective Mexican-hat like interaction and thus support the development of orientation maps. However, not all assumptions we made were in line with experimental evidence. In this section we will show, now only numerically, that one of these assumptions is not necessary, specifically that the addition of inhibitory to inhibitory connections does not prevent emergence of orientation maps.

To this end, we will use the exact GCAL model configuration that we have found in the previous section to possess the highest quality orientation map (see Figure 4). We will use the GCAL configuration in which we will explicitly model the inhibitory neurons (see Figure 3B, and Equation 10) and consequently also explicitly the excitatory to inhibitory and inhibitory to excitatory connections. Furthermore, we will add inhibitory to inhibitory connections to the model with the same extent as that of inhibitory to excitatory connections (σ_*i*_ = σ_*e*_ in Equation 10).

By explicitly modeling the inhibitory neurons in this model we have replaced a single parameter governing the strength of inhibitory lateral interactions in the model from the previous section with three new parameters that set: (1) the strength of excitatory to inhibitory, (2) inhibitory to excitatory, and (3) inhibitory to inhibitory connections (all other parameters remained the same as the in the best parameterization found in the previous section). Note that in principle, there is redundancy in these parameters as the overall strength of the projections from inhibitory neurons onto both excitatory and inhibitory populations is scaled by the excitatory to inhibitory projection strength. Therefore in Figure 5 we have systematically varied the strength of the added inhibitory to inhibitory projection expressed relatively to the strength of the inhibitory to inhibitory connections (which was set to 1), while also varying the strength of the excitatory to inhibitory projection. We have investigated the quality of the orientation maps that developed under these different levels of inhibitory to inhibitory interactions. As can be seen, high quality maps can develop under the full range of the inhibitory to inhibitory interaction strengths, depending on the overall excitatory to inhibitory drive. This shows that inclusion of direct inhibitory to inhibitory interactions does not invalidate the results of Section 3.1 (Figure 4).

### 3.3. Long-range excitation

In model variants 1 and 2 (Figures 4, 5) we have only assumed local connectivity by setting both excitatory and inhibitory interactions to have the same spatial extent. However experimental evidence shows that excitatory cells send longer connections compared to inhibitory cells (Budd and Kisvárday, 2001; Buzás et al., 2006). In this section we will explore what happens if we add long-range excitatory connectivity into model variant 1. Buzás et al. (2006) have shown that the lateral connectivity in layer 2/3 can be best described as superimposition of two gabor connectivity likelihoods, one short-range but not orientation specific and one long-range and orientation specific. Here we will assume such dual structure, leading us to add a second excitatory to excitatory and excitatory to inhibitory projection into model variant 1, but with a space constant that is 3 times larger (see Figure 3C), in line with Buzás et al. (2006) quantitative findings.

In Figure 6 we will examine what strength of the long-range excitatory connections, relative to the short-range excitatory ones (y axis in Figure 6A), leads to development of a high-quality orientation map. Adding the long-range excitation changes the spatial configurations over which excitatory interactions win over inhibitory ones, as well as the overall magnitude of the resulting net local excitation. Consequently, the proportion between the magnitude of the net local excitation due to the lateral interactions and the excitation due to afferent inputs is changed, which is a crucial parameter for map development. To compensate for these changes, we also systematically explore the ratio of the overall magnitudes of the lateral and afferent interactions (x axis of Figure 6). To make the parameter search computationally feasible, we perform the parameter search only in the region of parameters that allow for sufficiently strong long-range excitatory projections. As can be seen in Figures 6D,E, under appropriately strong lateral interactions, substantial long-range excitatory connections still permit the development of high quality orientation maps, demonstrating that the proposed model is consistent with the experimentally identified long-range excitatory connectivity.

### 3.4. Non-equal effective excitatory and inhibitory delays

In all model variants examined so far we have made the key assumption that the delay on the excitatory to excitatory connections is exactly equal to the sum of excitatory to inhibitory and inhibitory to excitatory delays. This assumption is approximately supported by the experimental evidence (Ohana et al., 2012), but we cannot assume it holds exactly in a real biological substrate. However, we hypothesize, that the small discrepancies between the delay of the mono-synaptic excitatory connections and the cumulative delay of the bi-synaptic inhibitory interactions can be absorbed into the membrane time-constant of the neurons. In this section we will verify this hypothesis by extending the modeling framework used thus far with a finite membrane time-constant (see Section 2) and proceed to determine the magnitude of the discrepancy in the delays between the excitatory and inhibitory interactions that can be managed by the model without impairing the resulting orientation map quality.

We use a model parameterization similar to those determined for model variant 1 (Section 3.1; Figure 3A). For simplicity and computational efficiency we omit the inhibitory to inhibitory and long-range excitatory connections that have already been investigated with model variants 2 and 3. The membrane time-constant of excitatory neurons was set to 2 ms while those of inhibitory neurons to 0.5 ms. These faster inhibitory dynamics are necessary to prevent oscillations in the system (Kang et al., 2003). We set the excitatory to excitatory delay to 1.4 ms and excitatory to inhibitory delay to 0.5 ms based on Ohana et al. (2012). In order to understand how closely the cumulative bi-synaptic inhibition delay has to match that of the direct excitatory to excitatory delay, we will vary the delay on the inhibitory to excitatory projection (note that we could achieve the same by varying the excitatory to inhibitory delays, and this choice was arbitrary).

**Figure 7:**
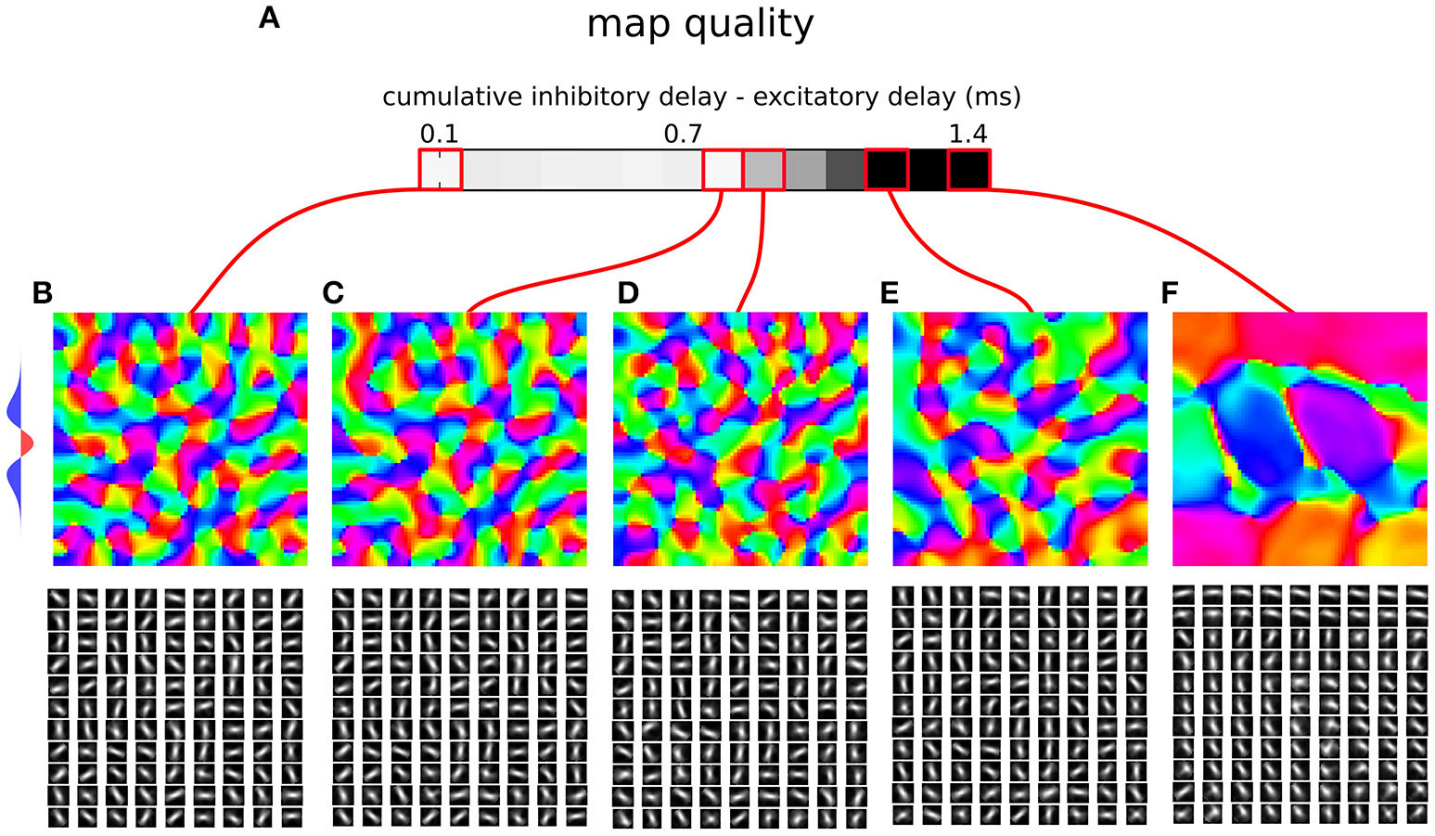
**Orientation map development in a rate model with a synaptic time constant. (A)** Map quality (see Section 2) at a range of cumulative inhibitory delays expressed as the difference from the direct excitatory to excitatory delays. **(B–F)** Functional organization in 5 parameter configurations indicated by the red marks. The orientation map (top), and afferent connection fields from the ON LGN model sheet for 81 example model V1 neurons (bottom). **(B–D)** Three examples of configurations where good quality orientation maps develop. **(E)** If the cumulative inhibitory delay is longer by more than approximately 0.8 ms in comparison to the direct excitatory delay the map quality starts to drop. **(F)** In the configuration corresponding to the case where delays on all connections are equal (i.e., the cumulative delays of the inhibitory interactions is twice as long as on the direct excitatory ones) orientation maps fail to develop. The lateral interaction kernel is indicated on the left, but note that it is valid only for the first parametrization, corresponding to equal delays on the mono-synaptic excitation and dy-synaptic inhibition pathway.

Figure 7 shows the resulting orientation maps and associated map quality measures of models with a range of differences between the delays of monosynaptic excitatory and bi-synaptic inhibitory interactions, that are in the figure expressed as the sum of the excitatory to inhibitory and inhibitory to excitatory delays minus the excitatory to excitatory delay (i.e., the figure shows how much slower the inhibitory disynaptic interactions were in comparison with the monosynaptic excitatory ones). As can be seen when the differences between the excitatory delay (1.4 ms) and cumulative inhibitory delay is small (<0.8 ms) high quality orientation maps develop in the model, confirming that sufficiently large discrepancy between the direct excitatory and bi-synaptic inhibitory delays can be accommodated in the model. However, as expected, as the difference between the delays increases the ability of the model to learn a topologically organized representation of orientation preference diminishes. Crucially, if the delays across all the projections were equal (Figure 7), as is typically assumed, the model fails to develop orientation maps in line with the analysis by Muir and Cook (2014), thus confirming that the specific delay pattern between neural types identified by Ohana et al. (2012) is key to achieving competitive dynamics in topologically organized neural models.

## 4. Discussion

In this study we have shown how recent findings on dependence of neural transmission delays on the type of pre- and post-synaptic neurons (Ohana et al., 2012) can resolve a long-standing question on how short-range inhibition can support cortical competition and consequently the development of functional cortical topological organization. Under simplifying assumptions, we have analytically shown how disynaptic inhibition that is as fast as mono-synaptic excitation can extend the effective range of inhibitory interactions, in contrast to the recent analytical results showing that in the case of equal synaptic delays on all connections the disynaptic inhibition has negligible effects (Muir and Cook, 2014). We have also shown that these findings are applicable to the problem of functional development in primary visual cortex. We have then proceeded to show using computational methods that the proposed models are robust to the addition of other well established features of cortical anatomy, commonly ignored by similar studies, including the long-range excitatory connections and mutual inhibition among inhibitory neurons. Finally, we have shown that the proposed mechanisms are robust to the variations of the exact delay ratio between the mono-synaptic excitation and di-synaptic inhibition. Overall, this study represents an important advance in our understanding of how orientation map development can be supported by the cortical neural substrate.

Using neuron reconstruction data of the recorded neurons, Ohana et al. (2012) identified the placement of the different pre-post synaptic combinations on the dendrites of the target cell as the likely origin of this transmission delay heterogeneity. They found that the excitatory-to-inhibitory synapses were closer to the soma of the post-synaptic neuron than the excitatory-to-excitatory and inhibitory-to-excitatory synapses. An axonal origin for the transmission delay heterogeneity is unlikely, as Ohana et al. (2012) found that all the pre-post combinations were, on average, equally distant from the pre-synaptic cell body. They further supported the dendritic origin of the observed delay inhomogeneities by showing in a computational model that the observed delay magnitudes can be explained by these anatomical findings. Overall this indicates a mechanism for the generation of the differences in inter-neuron transmission delays that is likely to generalize beyond the primary visual cortex. We thus suggest that the results presented in this study generalize to the development of other functional features and other cortical competition based mechanisms whose origin is outside of primary visual cortex.

**Figure 8:**
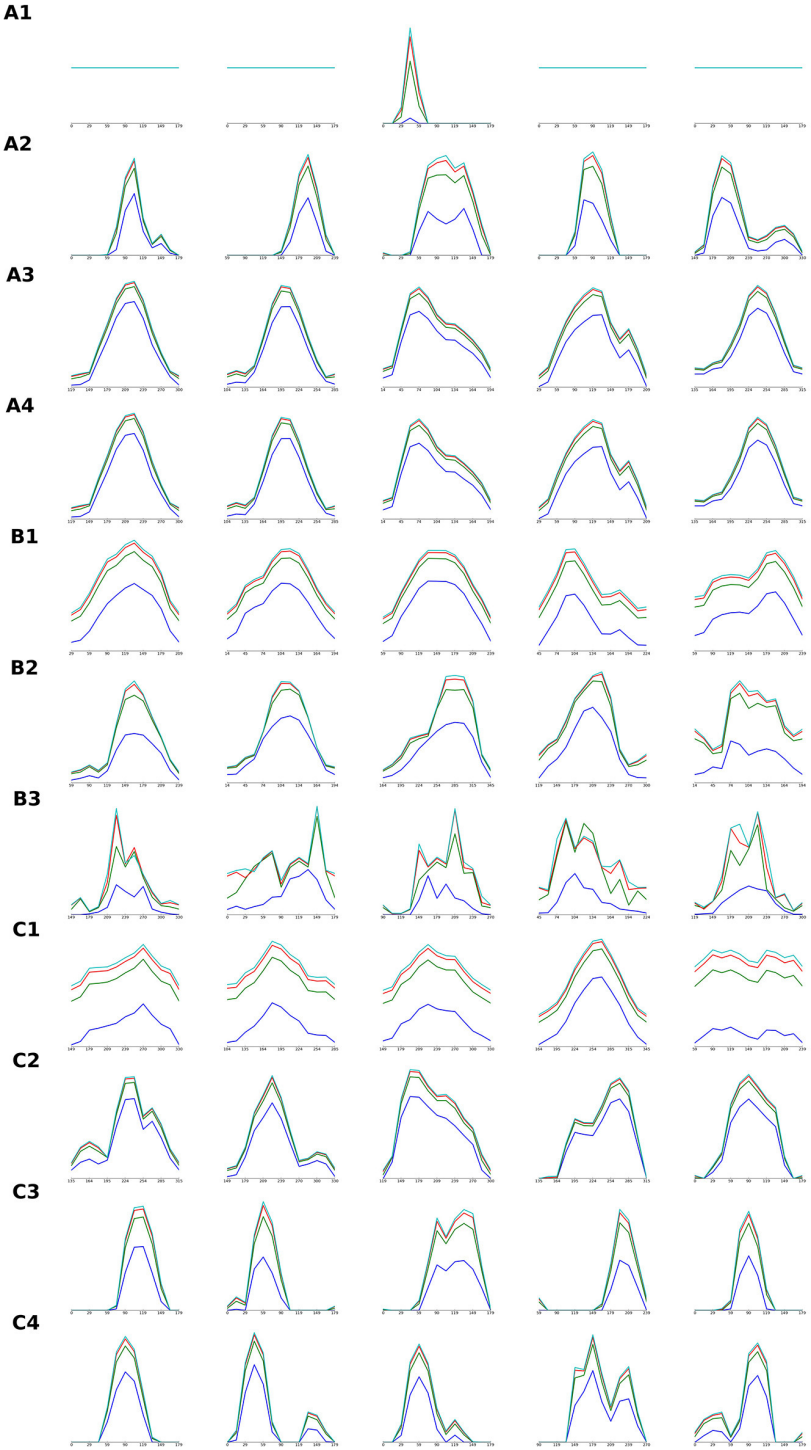
**Orientation tuning curves of individual V1 model units in models detailed in Figures 4–6**. Each row of the figure shows orientation tuning curves measured at 4 different contrasts of 5 model units, one in the center of the modeled cortical area and 4 positioned in the middle between the center and one of the 4 corners of the modeled cortical area. Each row corresponds to one of the model parameterizations that were detailed (i.e., their orientation map etc. was shown) in Figures 3–5: tuning curves marked with **(A)** correspond to models from Figure 4, **(B)** from Figure 5, and **(C)** from Figure 6. The order of the rows corresponding to a given Figures 4–6 (i.e., marked with **A,B,C**) is the same as the order in which the model parametrization in the given figure were presented (i.e., orientation tuning curves measured in the model parametrization presented in the 2nd row of Figure 5 will be displayed in the row marked as **(B2)** in this figure).

In this study we have focused on the development of the global organization of the thalamo-cortical and cortico-cortical connectivity and as a consequence the global organization of V1's most salient functional property—orientation tuning. How do, however, the individual model cortical units correspond to individual biological V1 neurons, e.g., do they have matching tuning properties? The detailed systematic investigation of this question is outside the scope of this study; however, to offer at least a basic view of how the explored models behave at single-cell level, we show in Figure 8 representative orientation tuning curves for all model parametrizations for which we have shown a detailed view of their properties (i.e., we have presented their orientation map etc.). Figure 8 shows that orientation tuning of individual neurons varies between the models and parametrizations, but all the models with good quality orientation maps also have reasonably realistic orientation tuning of single units. Several of these model parametrizations achieve sharp realistic contrast invariance of the orientation tuning width. At the same time, Figure 8 shows that the relationship between the orientation maps and afferent connectivity patterns and the orientation tuning is not straightforward. For example some parametrizations with inferior maps achieve sharper tuning (e.g., Figure 8, A2) in comparison with model parametrizations exhibiting high-quality orientation maps (e.g., Figure 8, A3). Ultimately, further, more rigorous, quantification and systematic investigation of the single cell tuning properties and their dependence on the model parametrization will be necessary to fully understand these relationships.

The most related past explanation of how cortical competition can arise under short range inhibition is that of Kang et al. (2003), who have shown that under the assumption of a faster inhibitory time constant (as opposed to excitatory), the effective excitatory and inhibitory interactions will follow the Mexican hat profile and thus support competition along the cortical surface. Indeed, in our final model variant 4 that explicitly considers membrane time-constant presented in Section 3.4, we assume that inhibitory neurons have faster membrane time constants than excitatory ones, as otherwise we observe oscillatory behavior in line with the analytical findings of Kang et al. (2003). Crucially, Kang et al. (2003) assumed instantaneous neural transmission, and when this biologically implausible assumption is rectified by addition of transmission delays that are uniform across the connections between the different pre- and post-synaptic neural types, we find that the competitive dynamics in the neural model break (Figure 7F) in line with the analytical and computational results of Muir and Cook (2014). However, when we replace the transmission delays with the neural-type specific pattern uncovered by Ohana et al. (2012) the competitive dynamics in the model are rescued and we observe development of high quality orientation maps (Section 3.4), in line with the analytical results under simplified conditions (Section 3.1).

The analytical results in this study were obtained only under simplifying assumptions, specifically instantaneous translation of inputs to membrane potential, equal extent of excitatory and inhibitory connections and a lack of inhibitory-to-inhibitory interactions. Even though we have shown computationally that these assumptions are not necessary for achieving the cortical competition and the consequential orientation map development sought in this study, further analytical work then can circumvent these simplifications would undoubtedly provide deeper understanding of the dynamics of the studied neural system and its dependence on the various parameters. This sentiment underlies the parameter explorations presented here, which show that even though the model is robust to changes in the considered parameters, the existence or not of dynamics supporting development of orientation maps can form a complex pattern within the explored parameter spaces. This is particularly the case for the model variant with inhibitory-to-inhibitory interactions, in which (unlike in the other variants) the inhibitory population gains it's own dynamics. Furthermore, the relative computational complexity of the studied models and the extensive set of parameters involved preclude systematic search across the full parameter space, and we have only explored parameters that we empirically found to have the biggest impact. Finally, one simplifying assumption that we have not treated in this study is the lack of direct thalamic input onto inhibitory cells. Since inhibitory cells in cortical layer 4 do receive thalamic input (Binzegger et al., 2004) the inclusion of external input in the inhibitory population needs to be considered in the future.

In this paper we have decided to investigate cortical competitive mechanisms through the prism of orientation map development. The advantage of this approach is that it allows us not only to show that some form of competition is possible, but also that it is of the form that actually supports implementation of specific cortical computations. Given that we show that our model implements effective Mexican hat lateral interactions (Section 3.1) and these have in the past been shown to be sufficient to explain cortical organization of other functional features (i.e., retinotopy, ocular dominance, spatial frequency and color) it is very likely that our results will generalize to these other dimensions of sensory input as well. Cortical competition of other forms has been proposed to underly a broad variety of other cortical operations, including associative memory, noise suppression, decision making, saliency detection and other forms of attentional computations. Even though additional work will be required to determine if the mechanisms proposed here can generalize to these other neural computations, this study offers a promising framework for anatomically plausible mechanistic explanations of these important aspects of brain function.

## Author contributions

JA performed the calculus, simulations, and wrote the manuscript.

## Funding

The research leading to these results has received funding from the European Union H2020 Programme (H2020-Adhoc-2014-20) under grant agreement no.720270 (The Human Brain Project SGA1).

### Conflict of interest statement

The author declares that the research was conducted in the absence of any commercial or financial relationships that could be construed as a potential conflict of interest.
